# Immune response after stimulation with wall components of Gram-positive bacteria

**DOI:** 10.1186/cc11942

**Published:** 2013-03-19

**Authors:** S Aloizos, E Tsigou, P Myrianthefs, S Gourgiotis, A Tsakris, G Baltopoulos

**Affiliations:** 1 NIMTS Hospital, Athens, Greece; 2 A. Anargiroi Hospital, Athens, Greece; 3 Medical School, Athens, Greece

## Introduction

The purpose of this study was to evaluate the immune response of patients susceptible to infection by Gram-positive bacteria after *ex vivo *provocation with lipoteichoic acid (LTA) and to compare the reaction with the one of healthy adults.

## Methods

Blood sample was obtained from 10 healthy volunteers, 10 hemodialysis patients with end-stage chronic renal failure (CRF), 10 patients with type II diabetes mellitus (DM) and 10 ICU patients on the second day of hospitalization, who suffered nonseptic SIRS and had an APACHE II score >25. After suitable treatment the samples were incubated with 1 mg LTA for 8 hours and maintained at -20°C until the measurement of cytokines TNFα, IL-6, IL-1β, and IL-10, using the ELISA method. The results are presented as mean values ± SEM. Graph Pad 4.0 was used, applying a *t *test to test the variation of each cytokine in each group, and ANOVA to assess the differences between the four groups.

## Results

Baseline cytokine values in the three groups were increased compared with the control group, but the difference was significant only for the ICU group (Table 1 data only for IL-6 and IL-10). The quotient IL-10/IL-6 of baseline values was between 0.23 and 0.96 among healthy, ESRD and DM persons, and 1.32 among ICU patients. In all examined groups the levels of cytokines increased significantly after stimulation with LTA, although ICU patients showed a differential response (a fivefold to ninefold rise compared with other groups who had an increase of 14-fold to 36-fold).

**Table 1:** Levels of cytokines before and after stimulation with LTA

	Control baseline LTA	ESRD baseline LTA	DM baseline LTA	ICU baseline LTA
IL-6	8.90 ± 0.76, 245.30 ± 26.68	86.60 ± 45.55, 1,310.00 ± 154.80	15.90 ± 1.89, 252.00 ± 35.52	372.40 ± 120.60, 3,659.00 ± 485.20
IL-10	3.00 ± 1.08, 40.90 ± 7.45	19.20 ± 7.14, 273.10 ± 126.50	15.30 ± 2.08, 350.50 ± 89.42	492.60 ± 66.72, 2,822.00 ± 432.70

Values in pg/ml.

## Conclusion

Severely ill patients and secondarily hemodialysis and diabetic patients are in a proinflammatory state. The response of all examined groups to provocation by LTA was sufficient, with a differential expression of severely ill patients, a fact that reffects their different immunologic status.

